# Is *Cryptococcus gattii* a Primary Pathogen?

**DOI:** 10.3390/jof1020154

**Published:** 2015-07-29

**Authors:** Kyung J. Kwon-Chung, Tomomi Saijo

**Affiliations:** 1 Molecular Microbiology Section, Laboratory of Clinical Infectious Diseases, National Institutes of Allergy and Infectious Diseases, NIH, Bethesda, MD 20892, USA; 2 Second Department of Internal Medicine, Nagasaki University Hospital, Sakamoto 1-7-1, Nagasaki-city, 851-8501, Japan; E-Mail: t-saijo@nagasaki-u.ac.jp

**Keywords:** Cryptococcosis, *Cryptococcus gattii*, anti-GM-CSF autoantibodies, immune dysfunction

## Abstract

The two etiologic agents of cryptococcal meningoencephalitis, *Cryptococcus neoformans* and *C. gattii*, have been commonly designated as either an opportunistic pathogen for the first species or as a primary pathogen for the second species. Such a distinction has been based on epidemiological findings that the majority of patients presenting meningoencephalitis caused by *C. neoformans* are immunocompromised while *C. gattii* infection has been reported more often in immunocompetent patients. A recent report, however, showed that GM-CSF (granulocyte-macrophage colony-stimulating factor) neutralizing antibodies were prevalent in the plasma of “apparently immunocompetent” *C. gattii* patients with meningoencephalitis. Because GM-CSF is essential for differentiation of monocytes to macrophages and modulating the immune response, it is not surprising that the lack of GM-CSF function predisposes otherwise healthy individuals to infection via inhalation of environmental pathogens such as *C. gattii*. Since the test for anti-GM-CSF autoantibodies is not included in routine immunological profiling at most hospitals, healthy patients with GM-CSF neutralizing antibodies are usually categorized as immunocompetent. It is likely that a comprehensive immunological evaluation of patients with *C. gattii* meningoencephalitis, who had been diagnosed as immunocompetent, would reveal a majority of them had hidden immune dysfunction. This paper reviews the relationship between GM-CSF neutralizing antibodies and the risk for *C. gattii* infection with CNS involvement.

## 1. Introduction

The etiologic agents of cryptococcosis, *Cryptococcus neoformans* and *C. gattii* are sibling species (85%–90% genomic identity) [1] that cause cryptococcosis in humans and a wide range of mammals. The disease in humans proceeds by inhalation of airborne cryptococci, which disseminate to the central nervous system (CNS) and cause menigoencephalitis. Unless treated, cryptococcal CNS infection is fatal and the fatality rate is high in spite of the most advanced therapeutic measures [2,3] Epidemiological studies have clearly shown that cryptococcosis due to *C. neoformans* is worldwide in distribution and though it can cause disease in otherwise healthy individuals, a majority of the reported cases have been from patients whose immune system have been compromised by various causes such as HIV infection, corticosteroid treatment and other immunosuppressive underlying conditions [3,4]. This is why *C. neoformans* has been widely known as an opportunistic pathogen. Epidemiological deviancy in *C. neoformans* infection, however, has been reported among patients in the Far East countries: China, Korea and Japan. In these countries, most of the cryptococcosis patients were caused by *C. neoformans* and were HIV sero-negative with or without any apparent predisposing underlying condition [5,6,7]. Cryptococcosis caused by *C. gattii* in HIV+ patients have been reported mostly in tropical and subtropical regions of the world such as Africa [8], South America [9] and Southern California [10].

Several studies have confirmed that *C. gattii* infects apparently healthy hosts with no known predisposing risk factor more frequently than immunocompromised individuals regardless of geographic regions and is therefore widely regarded as a primary pathogen [9,11,12,13,14,15,16]. While individuals with depleted CD4+ T cells resulting from HIV infection are highly susceptible to cryptococcal CNS infection due to *C. neoformans* [17,18], *C. gattii* infrequently infects such patients compared to *C. neoformans* even in the *C. gattii* endemic regions of the world [8,9,13,16]. Difference in host selection is not surprising considering their biological differences. The two species are readily distinguishable by the antigenic specificity of their polysaccharide capsule and their ability to utilize certain amino acids as carbon or nitrogen sources. *C. neoformans* includes strains of serotype A, D and AD which are generally unable to assimilate D-proline as a nitrogen source and glycine as a carbon source. *C. gattii* on the other hand can utilize D-proline as a nitrogen source and glycine as a source for both carbon and nitrogen [19,20]. These biochemical differences allow separation of the two species by using CGB agar media [19], which is commercially available (Hardy Diagnostics, Santa Maria, CA, USA; Thermo Fisher Scientific Remel Products, Lenexa, KS, USA). Recent studies have revealed further differences between the two species with regards to the utilization of D-amino acids as a nitrogen source in general [21] and the major target organs in mice [22].

It has been speculated that *C. gattii* infection in apparently healthy individuals is the result of increased environmental exposure to the fungus. Such speculation has been based on the over-representation of *C. gattii* infection among the Australian Aboriginal people living in rural areas [15,23] where daily exposure to the natural reservoir of *C. gattii* such as *Eucalyptus* and other trees, organic debris and soil is likely higher than the population living in urban areas. Analysis of the risk factors for *C. gattii* infection among patients in British Columbia, Canada showed that, in addition to environmental exposure, certain underlying medical conditions to be of significant risk [16]. These underlying medical conditions which carry a significant risk for *C. gattii* infection include preexisting oral steroid use, emphysema, chronic bronchitis, chronic obstructive pulmonary disease, sarcoidosis and pneumonia [16]. Environmental exposure risks included cutting/chopping wood, pruning and cleaning up branches [16].

The host specific mechanisms explaining the increased incidence of *C. gattii* infection in individuals with these risk factors have not been evaluated. Countless numbers of tourists and inhabitants are exposed to the environment of *C. gattii* endemic regions such as Vancouver, Canada, Southeast Asia and South America each year but the number of patients afflicted by *C. gattii* has only been a fraction of the exposed populations: less than one case per million per year in Australia [13] and Colombia [9] to 5.8 cases/million/year even in the highest case clusters reported in Vancouver Island, British Columbia during 1999–2007 [24]. This suggests that although the onset of *C. gattii* disease may be triggered by exposure, inhaled *C. gattii* cells in healthy individuals are readily eliminated by the host defense systems unless certain predisposing risk factors are present in these patients. Such endogenous risk factors may also contribute to increased persistence of dormant *C. gattii* or be required to trigger reactivation of dormant *C. gattii* to cause infection. GM-CSF neutralizing antibody appears to be one such host-dependent risk factor. Defining *C. gattii* as a primary pathogen may become obsolete as more subtle immune dysfunctions are recognized in otherwise healthy patients with *C. gattii* meningoencephalitis.

## 2. How Were GM-CSF Neutralizing Antibodies Identified as a Risk Factor for *C. gattii* Infection?

In 2013, Rosen *et al*., detected anti-GM-CSF autoantibodies which inhibited GM-CSF signaling in the plasma of four previously healthy HIV-negative patients suffering from cryptococcal meningoencephalitis [25]. This finding prompted them to screen archived plasma samples of 103 patients with culture proven cryptococcosis who had been treated at the National Institutes of Health between 1955 and 1984. Of the 103 archived plasma samples, 67 were from patients without any evidence of immunodeficiency and 36 were from patients with either a history of iatrogenic immunosuppression or an underlying medical condition that is known to predispose patients to cryptococcosis such as hematological malignancy or diabetes prior to their diagnosis of cryptococcosis. Of the 67 archived samples from patients without any recognized immunodeficiency, three were positive for the presence of anti-GM-CSF antibodies compared with none of the healthy controls (*n* = 64), diseased controls (*n* = 43, adult patients with undiagnosed immunodeficiency) or cryptococcosis patients with recognized immunodeficiency (*n* = 36). These results suggested that GM-CSF neutralizing antibodies could be a previously hidden risk factor for cryptococcal meningoencephalitis in HIV negative and otherwise healthy individuals. Of the seven cryptococcal strains (four from current and three from the archived cases) isolated from GM-CSF autoantibody positive patients, three had been reported as *C. neoformans*, one as *C. gattii* and three as *Cryptococcus* without identification at species level. The three *Cryptococcus* unspeciated strains had been isolated from the immunocompetent patients whose plasma samples had been archived and the strains had been maintained as lyophilized cultures.

Since the majority of cryptococcosis cases reported from Far East Asia are from HIV sero-negative patients with or without any apparent risk factors, we hypothesized that anti-GM-CSF autoantibodies could explain many of the cryptococcosis cases in otherwise healthy patients in Far East Asia [5,6,7]. As reported in 2014 [26], we screened 20 plasma samples from normal healthy volunteers and 21 otherwise healthy cryptococcosis patients from China with meningoencephalitis for the presence of functional anti-GM-CSF autoantibodies. To our surprise, GM-CSF neutralizing antibodies were detected in the plasma of only one patient who had been infected by *C. gattii* and one healthy volunteer, but none of the 20 patients infected by *C. neoformans* (Table 1) [26].

**Table 1:** Detection of anti-GM-CSF autoantibodies in plasma from Chinese immunocompetent, otherwise healthy cryptococcosis patients with CNS infection [26].

No. Patient Samples	Etiologic Agent	Anti-GM-CSF AB
20	*C. neoformans*, VNl, VNlll	0
1	*C. gattii*, VGl	1
20 (Normal volunteers)	None	1

**Figure 1:**
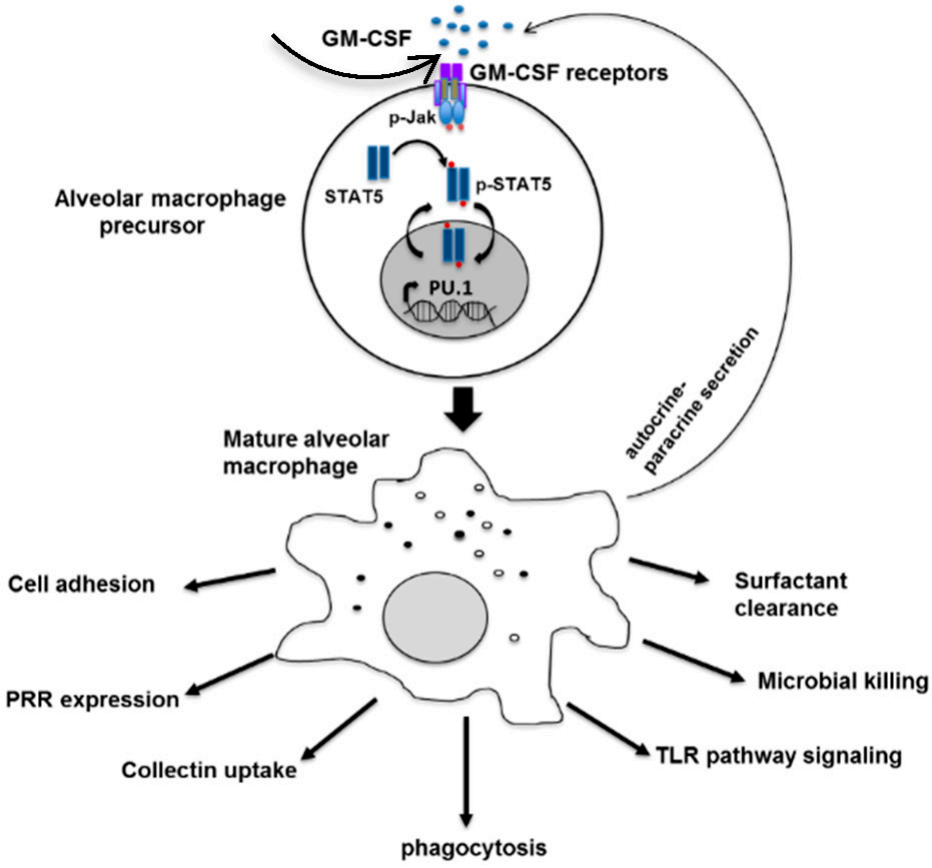
The role of GM-CSF in differentiation and innate immune function of the alveolar macrophage. Matured macrophages also secrete GM-CSF via autocrine and paracrine signaling. Adapted with permission from Shibata *et al.*, copyright Elsevier, 1982.

The *C. gattii* strain infected the GM-CSF autoantibody positive patients was of the VGI molecular type, which is the major *C. gattii* molecular type found in Southeast Asia [27]. The plasma from the *C. gattii* infected patient with GM-CSF autoantibodies inhibited phosphorylation of STAT5 (Figure 1) in normal PBMC when reacted with exogenous GM-CSF [26]. This indicated that the anti-GM-CSF autoantibodies in this *C. gattii* infected patient were biologically active. The GM-CSF autoantibody titer in the plasma of one healthy volunteer was significantly higher than the base level found in other healthy volunteers but lower than that of the *C. gattii* infected patient. Not surprisingly, the plasma of GM-CSF autoantibody positive healthy volunteer caused only partial inhibition of STAT5 phosphorylation compared to the plasma of the *C. gattii* patient with GM-CSF autoantibody [26].

This finding associating the GM-CSF autoantibodies with *C. gattii* infection but not with *C. neoformans* infection among Chinese patients (Table 1) prompted us to determine the species status of the three strains listed as *Cryptococcus* without species identification shown in Table 2.

**Table 2:** The etiologic agents of cryptococcosis reported in the seven patients with anti-GM-CSF autoantibodies by Rosen *et al*. [25] and the species confirmation by Saijo *et al*. [26].

Patient	1	2	3	4	5	6	7
Origin	S. CA ^a^	S. CA	Thailand	S. CA	NA ^b^	NJ ^c^	NJ
Infection	CNS/Lung	CNS/Lung	CNS/Lung	CNS/Lung Blood, Skin	CNS	CNS/Lung	CNS/Lung
Species	*C. neof.*	*C. neof.*	*C. neof.*	*C. gattii*	*Cryptococcus*	*Cryptococcus*	*Cryptococcus*
Species *Confirmation*	N.A ^d^	N.A	N.A	N.A	*C. gattii* VGlll	*C. gattii* VGl	*C. gattii* VGl

^a^ Southern California; ^b^ Information not available; ^c^ New Jersey, ^d^ Culture not available.

We found all three strains to be *C. gattii* strains of either VGlll or VGl molecular type [26]. This suggested that among the 67 apparently immunocompetent patients whose plasma had been archived, only three had been GM-CSF autoantibody positive and all had been infected by *C. gattii* while the antibodies were lacking in the plasma of the remaining 64 patients who had been infected by *C. neoformans.* The three strains originally reported as *C. neoformans* by Rosen *et al*. [25] but were not available to confirm their identification were most likely *C. gattii* instead of *C. neoformans*. They had originated either from Thailand or Southern California, regions endemic for *C. gattii* [28,29]. Most clinical laboratories designate the etiologic agents of cryptococcosis as *C. neoformans* without distinguishing between the two species. This is due to the application of the same therapy for both pathogens [30] and thus separation between the two species has not been widely practiced.

In order to confirm anti-GM-CSF autoantibodies as a higher risk factor for *C. gattii* CNS infection than for *C. neoformans* infection, we turned our attention to otherwise healthy *C. gattii* infected patients in Australia for two reasons. Australia is known for the high incidence of *C. gattii* infection in healthy host [13,15,23] and unlike the Chinese patients, Australian patients comprise multiple ethnic groups, which would eliminate any particular ethnicity-associated trait in the analysis. Nine plasma samples, eight from *C. gattii* infected and one from *C. neoformans* infected otherwise healthy Australian patients with meningoencephalitis were obtained and screened for GM-CSF autoantibodies. As shown in Table 3, six of eight (75%) otherwise healthy *C. gattii* patients had anti-GM-CSF autoantibodies in their plasma while the antibody was not detected from the immunocompetent patient with *C. neoformans* infection. These results clearly demonstrated that the GM-CSF neutralizing antibodies predispose healthy hosts to *C. gattii* infection.

Does this imply that GM-CSF neutralizing antibodies are a risk factor exclusively for infection due to *C. gattii* but not *C. neoformans*? Not likely since autoantibodies against GM-CSF have been known as a risk factor for other fungal infections, which are initiated by inhalation. Moreover, we have identified one *C. neoformans* VNl strain that had been isolated from a GM-CSF autoantibody positive, otherwise healthy cryptococcosis patient (unpublished data). The reason for higher association of GM-CSF autoantibodies with *C. gattii* infection than with *C. neoformans* infection is not clear. However, the higher prevalence of *C. gattii* than *C. neoformans* infection among patients with anti-GM-CSF autoantibodies could offer some clues to address the major differences in host’s immune responses to these two etiologic agents of cryptococcosis.

**Table 3:** Detection of GM-CSF autoantibodies in plasma of Australian immunocompetent otherwise healthy cryptococcosis patients with CNS infection [26].

Patients	Ethnicity	Etiologic Agent	Anti-GM-CSF Ab	Mol. Types
1	Caucasian	*C. gattii*	+	VGl
2	Caucasian	*C. gattii*	+	VGl
3	Caucasian	*C. gattii*	+	VGl
4	Aborigine	*C. gattii*	+	VGl
5	Caucasian	*C. gattii*	−	VGl
6	Asian	*C. neoformans*	−	NA
7	Asian	*C. gattii*	+	VGl
8	Caucasian	*C. gattii*	+	VGll
9	Asian	*C. gattii*	−	VGl

## 3. GM-CSF Neutralizing Antibody and Susceptibility to Cryptococcal Infection

Granulocyte-macrophage colony stimulating factor (GM-CSF) is one of the family of glycoprotein cytokines that mediates the survival, proliferation, differentiation and function of hematopoietic cells [31,32,33]. In human and mice, GM-CSF is involved in terminal differentiation of monocytes to alveolar macrophages [34,35], regulation of neutrophil functions [36], differentiation of dendritic cells [37] and augments innate immunity mostly through PU.1 [38,39] (Figure 1). The importance of GM-CSF in host defense against *C. neoformnans* has been recognized since the early 1990s when GM-CSF neutralizing antibody increased mortality of *C. neoformans* infected mice with rapid progression of meningoencephalitis [40]. Corroborating this observation, GM-CSF knockout mice (GM^−/−^ mice) failed to clear the fungus, which led to higher cryptococcal burdens in pulmonary cryptococcosis because GM-CSF was required for early influx of macrophages, CD4 and CD8 cells into the lung [41]. Additionally, GM-CSF enhanced the anticryptococcal activity of human monocytes, neutrophils and macrophages and showed a synergistic effect with azoles suggesting a therapeutic implication for cryptococcosis [42,43].

Furthermore, cryptococcosis is reported more frequently than other fungal disease in patients with acquired pulmonary alveolar proteinosis (PAP) [44]. When autoantibodies block the GM-CSF pathway, differentiation and function of alveolar macrophages are impaired and the ability to clear surfactants is diminished [44,45,46] (Figure 1). As a result, pulmonary alveoli eventually accumulate periodic acid-Schiff (PAS)-positive proteinaceous surfactant components [44,47,48]. This feature is characteristic of acquired pulmonary alveolar proteinosis (PAP) originally termed by Rosen *et al*., as “idiopathic PAP” [49]. This differentiates the acquired PAP from the primary PAP, which results from mutations in the genes encoding the GM-CSF receptor [50], surfactant protein B or C, and from the secondary PAP. Secondary PAP develops in association with functional impairment or reduced number of alveolar macrophages such as due to some hematologic cancers, inhalation of inorganic dusts or toxic fumes and pharmacologic immunosuppression [51]. While idiopathic PAP was eventually identified as an autoimmune disease caused by GM-CSF autoantibodies [51,52,53], the primary stimulus leading to the production of GM-CSF autoantibodies remains unknown [54,55]. To advance the understanding of PAP pathogenesis, exogenous GM-CSF was administered to patients with acquired PAP, which appeared to benefit patients with therapeutic efficacy [56,57].

Since autoantibodies to GM-CSF cause defects in chemotaxis, adhesion, phagocytosis, microbicidal activity and phagolysosome fusion of alveolar macrophages [51], patients with PAP are at risk for infections from a variety of respiratory microorganisms including fungal species [25,44,58,59]. Among the 27 acquired PAP patients first reported by Rosen in 1958, one (patient 24) had been infected by *Cryptococcus*. The etiologic agent of cryptococcosis in this patient could have been *C. gattii* rather than *C. neoformans*. The patient was working in a lumberyard in Massachusetts when he was diagnosed with cryptococcosis [49]. The patients were likely exposed to high concentrations of wood dust. Massachusetts is not known as a region endemic to *C. gattii* but the lumberyard could have been handling trees imported from regions where *C. gattii* is endemic. This case is reminiscent of the first autochthonous case of *C. gattii* infection in Germany in an apparently immunocompetent healthy male who had been working in sawmills and woodworking factories and exposed to high levels of wood dust, including that of imported tropical trees [60]. This case report had appeared at least five years prior to the first discovery of *Eucalyptus* trees as the environmental source of *C. gattii* in Australia [61].

Although less frequently reported than cryptococcosis [25,26,49,62,63,64,65], a literature survey revealed five other fungal pathogens that caused infection in patients with acquired PAP. They are all mycoses controlled by macrophages upon inhalation: four cases due to *Aspergillus fumigatus* [58,59,66,67,68], one possible *Blastomyces dermatitidis* case [69], one *Coccidioides* case [70], one Mucorales case [48] and four cases due to *Histoplasma capsulatum* [49,71]. GM-CSF is reported as a critical cytokine in the generation of an optimal protective immune response against pulmonary challenge of mice with *H. capsulatum* [72]. Neutralization of GM-CSF resulted in an increase in fungal burden and higher mortality suggesting that the GM-CSF enhances antimicrobial defenses against intracellular pathogens such as *H. capsulatum*. The mode of anti-*Histoplasma* action enhanced by GM-CSF in mice was reported as the sequestration of labile Zn in infected macrophages by inducing metallothioneins in a STAT3 and STAT5 dependent manner [73,74]. Since Zn is a basic element essential for organismal growth, Zn deprived *Histoplasma* fails to replicate within the macrophages leading to fungal clearance [73,74]. GM-CSF also promoted resistance to *Aspergillus c*onidia by maintaining proinflammatory response by alveolar macrophages [75].

Why do GM-CSF autoantibodies pose a higher risk for *C. gattii* than *C. neoformans* infection? Before attempting to address this question, it is necessary to investigate whether anti-GM-CSF autoantibodies are also prevalent in pulmonary *C. gattii* infection without CNS involvement since evaluation of GM-CSF autoantibodies and cryptococcosis thus far has only focused on patients with CNS infection. Pulmonary infection without CNS involvement is more prevalent with *C. gattii* than with *C. neoformans* infection [12,16,76]. If GM-CSF autoantibodies are prevalent only among the *C. gattii* patients with CNS involvement, it suggests that GM-CSF may play a more important role in dissemination of *C. gattii* than *C. neoformans* to the brain from the lung and/or brain immunity against *C.gattii* than *C. neoformans*. Little is known about the role of GM-CSF in dissemination of *Cryptococci* to the brain or brain immunity against the fungus. Our study with GM-CSF^−/−^ mice suggested that a lack of the cytokine function enhances dissemination of both the *C. neoformans* strain H99 and the *C. gattii* VG1 strain isolated from the GM-CSF antibody positive Chinese patient [26] from the lung to the brain (unpublished data). However, the effect was significantly greater with the VGI strain compared to H99. Investigation of the immunological mechanism responsible for this difference between dissemination of H99 *vs.* the VGI strain is currently underway in our laboratory and should shed more light as to the role of GM-CSF in dissemination and brain immunity against *C. gattii*. Although GM-CSF does not appear to influence brain microglial killing of *C. neoformans* serotype A strains [77], there has not been any study on killing of *C. gattii* by immune cells in the brain.

## 4. Could *C. gattii* Infection Have Induced Production of GM-CSF Neutralizing Antibodies?

Before GM-CSF autoantibody was known as the cause of PAP, a hypothesis that PAP is an abnormal pulmonary response to an unusual infectious agent such as *Pneumocyctis carinii* [49,78,79] (now called *P. jirovecii*) or *C. neoformans* [80] gained some support [44]. However, the lung lavage fluids of a vast majority of PAP patients have been found to be free of pathogens and it has been recognized that most infectious cases in PAP patients are secondary in origin [44]. Among the first 27 cases of PAP patients reported, there were only two patients with superimposed fungal infections (one case each of histoplasmosis and cryptococcosis), which indicates that production of GM-CSF autoantibodies resulting in PAP was not due to fungal pathogens [49]. In the case of the seven GM-CSF autoantibody positive cryptococcosis patients reported by Rosen and colleagues [25] as well as the seven patients reported by Saijo *et al*. [26], none of them were diagnosed with PAP at the presentation of cryptococcal CNS infection. Only one patient developed symptomatic PAP two years later and another patient developed radiographic and cytopathologic changes without symptoms [25]. This suggests that the GM-CSF autoantibody positive cryptococcosis patients could eventually develop PAP but that cryptococcosis was not the cause of PAP. This also suggests that PAP symptoms were not necessary for GM-CSF autoantibody-associated *C. gattii* infection. There is a possibility that pulmonary infection with *C. gattii* stimulates GM-CSF production more than *C. neoformans* and the high cytokine production by *C. gattii* for a long period of time can cause induction of functional antibodies against the cytokine. Could *C. gattii* also exacerbate the production of GM-CSF autoantibodies more than *C. neoformans* in the patients who already are antibody positive? This question cannot be answered since the anti-GM-CSF antibody titers in these patients prior to *C. gattii* infection are unknown and there are no longitudinal studies on the GM-CSF autoantibody titers during the infection in such patients. Monitoring the health status and the GM-CSF autoantibody titers over time in the healthy Chinese volunteer positive for the cytokine antibodies [26] could offer valuable information concerning this question.

## 5. Conclusions

Anti-GM-CSF autoantibodies are apparently an important underlying risk factor for meningoencephalitis caused by *Cryptococcus* in otherwise healthy individuals. For some unknown reason, the neutralizing antibodies against GM-CSF appear to be a greater risk for cryptococcosis due to *C. gattii* than *C. neoformans.* This etiologic agent’s difference in the prevalence of GM-CSF neutralizing antibodies among otherwise healthy cryptococcosis patients may offer an important clue in order to address the differences in the host’s immunological response to the two pathogens. It is important to also include the GM-CSF autoantibody test during immunological profiling of cryptococcosis patients who appear to be otherwise healthy. This is not only for the assessment of subtle underlying immune disorders but also for better clinical management for those positive for GM-CSF antibodies since they may eventually develop PAP after meningoencephalitis.
