# Correction: Kwon-Chung, K.J. et al. Is *Cryptococcus gattii* a Primary Pathogen? *J. Fungi* 2015, *1*, 154–167

**DOI:** 10.3390/jof2030020

**Published:** 2016-07-05

**Authors:** Kyung J. Kwon-Chung, Tomomi Saijo

**Affiliations:** 1 Molecular Microbiology Section, Laboratory of Clinical Infectious Diseases, National Institutes of Allergy and Infectious Diseases, NIH, Bethesda, MD 20892, USA; 2 Second Department of Internal Medicine, Nagasaki University Hospital, Sakamoto 1-7-1, Nagasaki-city 851-8501, Japan; t-saijo@nagasaki-u.ac.jp

The authors of the published paper [1] would like to correct Table 1. The third line in the first column should have been 20 (normal volunteers) and the etiologic agent in the second column should have been “None” instead of *C. gattii*, VGI. Therefore, Table 1 should read as follows: 

We apologize for any inconvenience caused to readers.

## Figures and Tables

**Table 1:** Detection of anti-GM-CSF autoantibodies in plasma from Chinese immunocompetent, otherwise healthy Cryptococcosis patients with CNS infection [26].

No. Patient Samples	Etiologic Agent	Anti-GM-CSF AB
20	*C. neoformans*, VNl, VNlll	0
1	*C. gattii*, VGl	1
20 (Normal volunteers)	None	1
